# Single-cell and spatial sequencing identifies senescent and germinal tumor cells in adamantinomatous craniopharyngiomas

**DOI:** 10.1186/s13578-024-01299-1

**Published:** 2024-09-02

**Authors:** Xianlong Wang, Jincheng Lin, Hongxing Liu, Chuan Zhao, Zhiwei Tu, Dapeng Xu, En Zhang, Zhongqing Zhou, Xueling Qi, Xingfu Wang, Zhixiong Lin

**Affiliations:** 1 Department of Bioinformatics, School of Medical Technology and Engineering, Key Laboratory of Ministry of Education for Gastrointestinal Cancer, Fujian Medical University, Fuzhou, China; 2 Department of Neurosurgery, Sanbo Brain Hospital, Capital Medical University, Beijing, China; 3 Department of Neurosurgery, Beijing Ditan Hospital, Capital Medical University, Beijing, China; 4 Department of Neuro-Oncology, Sanbo Brain Hospital, Capital Medical University, Beijing, China; 5 Department of Pathology, Sanbo Brain Hospital, Capital Medical University, Beijing, China; 6 Department of Pathology, The First Affiliated Hospital of Fujian Medical University, Fuzhou, China

**Keywords:** Adamantinomatous craniopharyngioma, Single-cell sequencing, Spatial sequencing, CTNNB1, Heterogeneity

## Abstract

Adamantinomatous craniopharyngioma (ACP) is a clinically aggressive tumor without effective treatment method. Previous studies proposed a paracrine tumorigenesis model, in which oncogenic β-catenin induces senescence in pituitary stem cells and the senescent cells lead the formation of paracrine tumors through secretion of pro-tumorigenic factors. However, there lacks characterization on senescent cells in ACPs. Here, we profiled 12 ACPs with single-cell RNA and TCR-sequencing to elucidate the cellular atlas in ACPs and 3 of them were also subject to spatial sequencing to localize different subpopulations of the tumor cells. In total, we obtained the transcriptome profiles of 70,682 cells. Tumor cells, which were unambiguously identified through the cellular mutation status of the driver *CTNNB1* mutations, were clustered into 6 subsets. The whorl-like cluster (WC) cells show distinct molecular features from the other tumor cells and the palisading epithelium (PE) cells consists of a proliferating subset. Other than typical PE and WC, we identified two novel subpopulations of the tumor cells. In one subpopulation, the cells express a high level of cytokines, e.g., *FDCSP* and *S100A8*/*A9*, and are enriched with the senescence-associated secretory phenotype (SASP) factors. Hematoxylin and eosin staining reveals that these SASP cells lack an ordered structures and their nuclei are elongated. In the other subpopulation, the cell sizes are small and they are tightly packed together with an unusual high density expressing a high level of mitochondrial genes (median 10.9%). These cells are the origin of the tumor developmental trajectories revealed by RNA velocity and pseudo-time analysis. Single-cell RNA and TCR analysis reveals that some ACPs are infiltrated with clonally expanded cytotoxic T cells. We propose a hypothesis that WC and PE are formed via different negative regulation mechanisms of the overactivated WNT/β-catenin signaling which provides a new understanding on the tumorigenesis of ACPs. The study lays a foundation for future studies on targeting senescent cells in ACPs with senolytic compounds or other therapeutic agents.

**Supplementary Information:**

The online version contains supplementary material available at 10.1186/s13578-024-01299-1.

## Introduction

Adamantinomatous craniopharyngioma (ACP) is an intracranial tumor localized within the sellar and parasellar region with complex histopathological structure. It may arise from squamous epithelial cell nests occurring anywhere along the craniopharyngeal duct [13, 20, 25]. ACP represents a relatively small but clinically significant number of pediatric brain tumors although it is diagnosed in both children and adults [25]. Adjacent to the pituitary gland, hypothalamus and optic chiasm, it is associated with high morbidity and occasional mortality [28, 30, 40]. It often forms finger-like protrusions at the interface between tumor and brain tissues and invades the important neural structures around the hypothalamus, causing extensive neurological damage and poor prognosis [3, 23, 30, 32]. ACP is primarily treated by surgery and radiotherapy. However, gross resection is not only challenging but also with a high risk of recurrence and postoperative sequalae [32]. Despite the development of microsurgical techniques in recent years, the prognosis of ACP remains unsatisfactory, the recurrence rate is still high and the effect of drug treatment is not good [30, 32, 40]. Therefore, it is of great significance to gain deeper understanding on the cell types and biological characteristics of ACP for developing better clinical treatment.

Although it has been established that ACP is driven by abnormal activation of the WNT/β-catenin signaling pathway due to nuclear-accumulation of degradation-resistant β-catenin as a result of *CTNNB1* exon3 mutations, the drug development effort to inhibit this pathway is not successful yet [11]. Response to intralesional and systemic administration of interferon α was reported in case studies, but a phase II clinical trial has failed [8]. Novel treatments are needed to develop against these childhood neoplasms based on more comprehensive understanding of the pathogenesis. Murine models suggest a paracrine tumorigenesis model where β-catenin accumulating tumor cells secret senescence-associated factors, e.g., SHH, FGFs, BMPs, proinflammatory cytokines and chemokines to drive tumor growth and activate immune response [2, 3]. However, the hypothesis needs to be confirmed in human ACPs and the communication signals remain to be elaborated between tumor cells and other cells in the tumor microenvironment. If the hypothesis is validated, targeting senescent cells will be a potential therapeutic strategy for ACPs.

Histologically, ACPs are complex tumors consisting of solid, cystic and calcified components and cholesterol clefts. The tumors often show local invasion of the surrounding brain tissue. Typical features include β-catenin nuclear-accumulated whorl-like clusters, palisading epithelium (PE), stellate reticulum (SR), wet keratin (ghost cells) nodules, cysts and calcifications [3, 23, 42]. It remains unclear how this complex histopathological structure is developed and what molecular characteristics are in each component. Apps et al. used laser capture microdissection (LCM) and RNA-sequencing technique to profile the clusters, PE and glial reactive tissue to characterize molecular signatures in each structure [3]. With the development of single-cell transcriptomics in recent years, researchers have conducted in-depth studies on the tumor microenvironment of ACPs [1, 12, 29]. At the resolution of single cells, Jiang et al. have found that the cell types in the ACP tumor microenvironment include tumor epithelial cells, immune cells, and neuroglial cells [12]. Among them, tumor epithelial cells are divided into four states based on gene expression characteristics: whorl-like epithelium (WE), PE, keratinized-like epithelium (KE), and cycling cell (CC). In addition, a group of oligodendrocyte lineages related to immune infiltration and nerve damage were also found in ACP.

These findings reveal ACP’s complex histological structure and biological behavior, providing new perspectives for understanding their pathogenesis and treatment. However, further research is needed to further explore the pathological mechanisms in order to develop new targets and strategies for precise diagnosis and effective treatment of ACP. First of all, the sample size in previous LCM or single-cell RNA sequencing studies is limited, which makes it impossible to deeply analyze the inter-tumoral heterogeneity. Our previous study and others’ studies with bulk RNA sequencing and DNA methylation profiles have identified a large degree of inter-tumoral heterogeneity and the existence of molecular subgroups in ACPs which is significantly different from the situation in papillary craniopharyngiomas (PCPs) driven by *BRAF* V600E mutations [37]. A limited sample size may also prevent one to identify all kinds of cells in the tumor microenvironment. For example, in Jiang’s study, no fibroblasts or other stromal cells were reported [12]. There is also a lack of spatial transcriptome data, which makes it impossible to analyze the spatial distribution and interactions of tumor cells and other types of cells in the tumor microenvironment. Although the molecular features have been depicted for the whorl-like and palisade-like epithelial cells, there still lacks an understanding on the tumor germinal center, molecular characteristics of squamous epithelium, stellate reticulum, and wet keratin nodules and these are typical histological features of ACPs. Furthermore, the developmental trajectory and formation mechanism is still unclear among these tumor components.

The aim of this study is to further expand the sample size and conduct in-depth research on ACP by combining single-cell and spatial transcriptome technologies on the basis of our previous bulk RNA sequencing study [37]. The specific objectives include: to analyze the heterogeneity of ACP tumor cells and identify novel tumor cell subpopulations; to elucidate the evolutionary relationships of ACP tumor cells and identify key regulatory factors; to characterize the spatial distribution and interactions of tumor cells and to construct a tumor microenvironment atlas. This study will provide a scientific basis for the development of new targets and strategies for diagnosis and treatment of ACP.

## Materials and methods

### Patients and specimen collection

The study was reviewed and approved by the human subjects’ institutional review boards at Sanbo Brain Hospital (SBNK-YJ-2020–014-01) and Fujian Medical University (FMU-2019–43) and in accordance with the regulations of the Declaration of Helsinki. Written informed consent was obtained from all patients or their guardians. Tumor specimens were obtained from the resection surgery. Histological diagnosis was reconfirmed on all tumors by neuropathologists. Fresh tumor samples from 12 ACPs were collected for single-cell sequencing and 3 of them were also subject to spatial transcriptome sequencing (Suppl. Table 1). None of the patients was treated with chemotherapy or radiation prior to tumor resection.

### Single-cell sample preparation and sequencing

The calcified tissues and burned tissues from the resection surgery were removed from the tumor specimens. The rest of the tissue was rinsed with 0.9% saline or 1 × DPBS, then cut into small pieces to prepare the single-cell suspension. According to the tissue size, 2.5–5 ml collagenase I (1 mg/ml) was added to sufficiently digest the tissue for 90 min. The cell suspension was filtered through a 70 μm cell strainer and centrifuged for 7 min at 300 *g*. The sediment was washed twice with precooled 1 × DPBS containing 0.04% BSA after removing the supernatant. Dead cells were eliminated by excluding Sytox‐positive cells (SYTOX Blue dead cell stain, Miltenyi Biotec, CA, USA) according to the manufacturer's instructions. The quality control of the cell suspension was estimated using a Countess II Automated Cell Counter.

The cell suspension was loaded into Chromium microfluidic chips with 3′ v3 chemistry and barcoded with a 10 × Chromium Controller (10X Genomics). RNA from the barcoded cells was subsequently reverse-transcribed and sequencing libraries constructed with reagents from Chromium Single Cell 5′ v3 Reagent kit, Single Cell V(D)J Enrichment kit (10X Genomics) according to the manufacturer’s instructions. Sequencing was performed with Illumina NovaSeq 6000 according to the manufacturer’s instructions (Illumina).

### Single-cell RNA sequencing data processing

Reads from each sample were processed with CellRanger (v3.0.1) pipeline separately with human genome GRCh38 reference. The filtered count matrices were loaded into Seurat (v5.0.1, [10]) and Monocle3 (v1.3.1, [34]) for downstream analysis, including data normalization, dimensionality reduction, clustering and differential expression. The results obtained from Monocle3 are presented in the Results section. Secondary filtering was performed by Seurat to remove the cells with no more than 50 genes expressed, those with more than 25% mitochondrial genes or more than 1% hemoglobin genes, and the genes with expression in fewer than 3 cells. The DoubletFinder (v2.0.3, [24]) algorithm was used to filter out the doublet profiles. Multiple profiles were integrated with Harmony (v0.1.0, [14]) in Seurat.

Normalization was processed with the principal component analysis (PCA) and 250 principal components were retained in Monocle3. Dimensionality reduction was performed with the Uniform Manifold Approximation and Projection (UMAP) algorithm for visualization. Cell clustering was carried out with Levine et al.’s phenoGraph algorithm [17]. Similar clusters were merged together into larger, better separated groups called ‘partitions’ using Wolf et al.’s PAGA algorithm [38]. Marker genes expressed by each cluster were identified with the ‘top_markers’ function and sorted according to the ‘pseudo-R2’ metric.

Cells were annotated with the aid of SingleR (v1.2.4, [4]) using BlueprintEncodeData and HumanPrimaryCellAtasData gene sets and confirmed with expression plots of marker genes from the CellMarker database (http://bio-bigdata.hrbmu.edu.cn/CellMarker/) and literature. Cell cycle phases were scored with ‘CellCycleScoring’ function in Seurat.

### Spatial sequencing and data processing

Formalin-fixed paraffin-embedding (FFPE) blocks were cut into 6.5 × 6.5 mm sections and processed using Visium Spatial Gene Expression Kit (10X Genomics) according to manufacturer’s instructions. First, tumor tissue permeabilization condition was optimized using Visium Spatial Tissue Optimisation kit. Sections were H&E stained and imaged using a Leica microscope DM6000 (Leica, DE) under a 20 × lens magnification, and the sections with typical ACP histopathological characteristics were chosen and processed for spatial sequencing. The resulting cDNA library was checked for quality control, then sequenced on an Illumina NovaSeq 6000 (Illumina). The spots were manually annotated by a specialist neuropathologist using Loupe (v4.0.0, 10X Genomics).

Reads from each sample were processed with SpaceRanger (v1.3.0) pipeline separately to obtain the feature-spot matrices which were loaded into Seurat for downstream analysis. The probabilistic transfer method based on the ‘SCTransform’ normalization algorithm in Seurat was used to predict the major cell type in each spot using the annotations of scRNA profiles as the reference.

### Functional enrichment analysis

Marker genes in each ‘cluster’ or ‘partition’ were identified with the ‘top_markers’ function in Monocle3. Differential expression analysis was also performed using FindMarkers in Seurat with the Wilcoxcon rank sum test and the RankCompV3 algorithm [39]. The AUCell algorithm implemented with Julia was used to identify cells with active gene signatures.

### Transcriptional dynamics and trajectory analysis

RNA velocity analysis in single-cell RNA-seq profiles was performed with scVelo (v0.2.5) with the dynamical model [5]. Pseudo-time trajectory analysis of single-cell profiles was performed with Moncole2 (v2.16.0).

### V(D)J data analysis

Reads were processed with CellRanger (v3.1.0) vdj function for alignments and contig annotations. T cell receptor (TCR) repertoire analysis was performed with ScRepertorie (v1.3.5, [6]) by integrating single-cell profiles and TCR contigs.

### Cellular *CTNNB1* mutation status and tumor cell annotation

From the reads-alignment BAM files of scRNA-seq data, *CTNNB1* exon 3 reads were extracted and analyzed for the mutation status in each cell with an in-house package (https://github.com/pathint/scSNVcaller). The algorithm compared the bases at the given mutation sites, which were known from the whole-exome sequencing (WES) or bulk RNA-seq, with those in the reference and the variant sequences. Each cell is classified as mutated (at least one read carrying the known variant allele), wildtype (carrying the reference allele) or unknown (no reads covering the mutation site). Spatial RNA sequencing data and RNA profiles from the laser capture microdissection (LCM) specimens of 3 whorl-like clusters and 3 PE regions (SRA: ERP019971) were compared with the scRNA-seq data to further confirm the pathological origin of different subsets of tumor cells.

### Transcription factor analysis

Gene regulatory networks was constructed from single-cell RNA-seq data with pySCENIC (v. 0.10.4) through the VSN-pipeline. The GENIE3 algorithm was first used to generate co-expression gene regulatory networks (GRN), and the co-expression data was then subjected to cis-regulatory motif analysis using the RcisTarget algorithm. Furthermore, the AUCell algorithm was used to score the activity of significant regulons enriched in different clusters.

### Immunostaining of histological sections

Immunohistochemistry staining was performed as previously described [37]. Antibodies include β-catenin (ZM-0442, 1:200, Zhongshan Goldenbridge Biotechnology, Beijing, China), LIF (K002699P, 1:500, Solarbio Science & Technology Co., Beijing, China). IHC results from the previous study [37] were reanalyzed to compare the immune infiltration between the relapsed and the primary tumors.

## Results

### Cellular map of ACPs shows significant inter-tumoral heterogeneity

To generate a cellular map of ACP tumors, we used single-cell RNA (scRNA-seq) and T cell receptor (TCR) sequencing (scTCR-seq) to profile surgically resected fresh tumors from 12 ACP patients, among which 7 are primary tumors and the other 5 are relapsed tumors (Suppl. Table 1). The ages at the initial diagnosis range from 3 to 50 years old. To localize the spatial distribution of different types of the cells in the tumor microenvironment, we also used the Visium Spatial Transcriptomics technology to profile 3 formalin-fixed paraffin embedded (FFPE) tumor tissue sections out of the 12 ACPs.

**Fig. 1:**
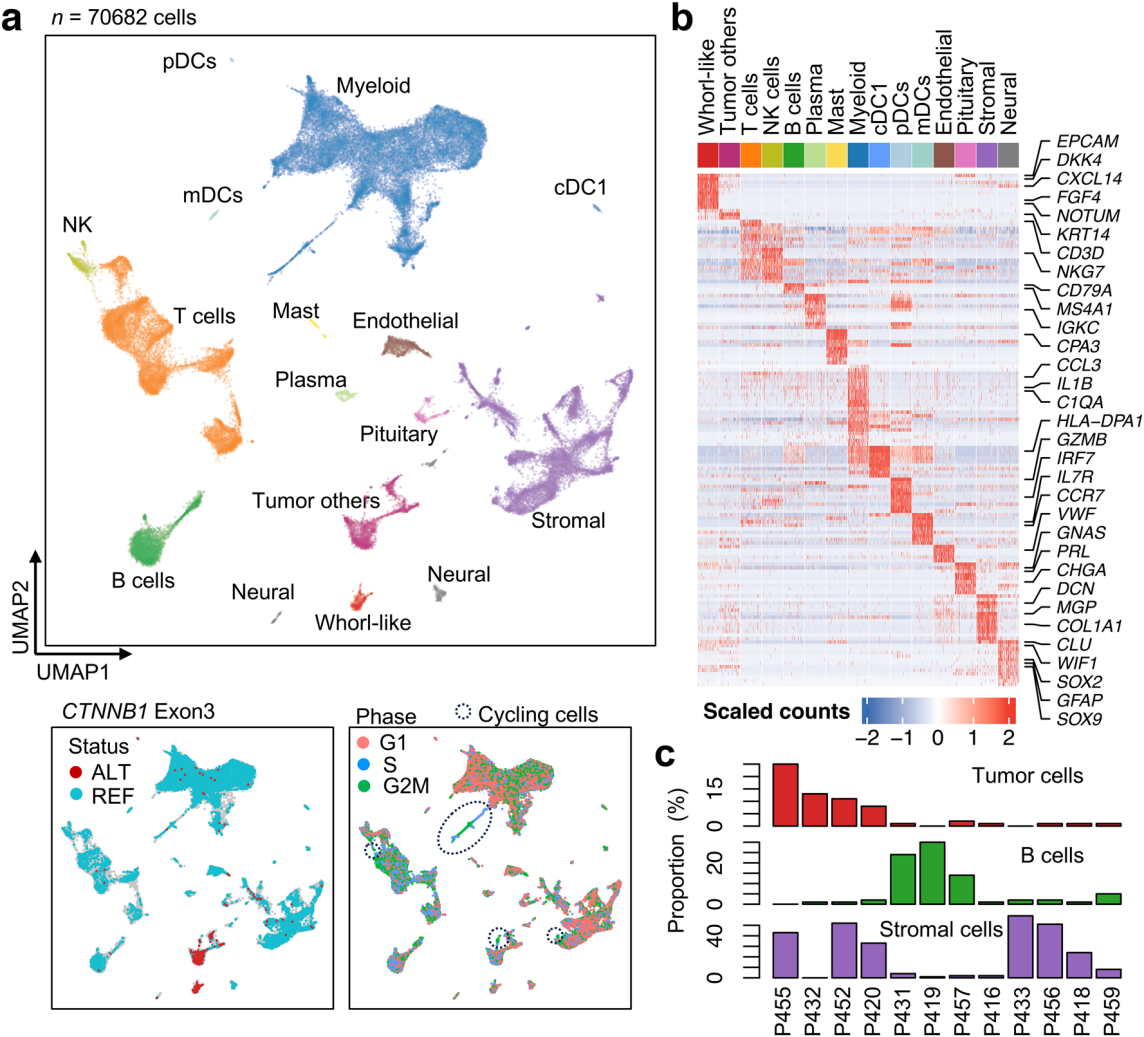
Single-cell transcriptomic profiling of adamantinomatous craniopharyngiomas revealing intra- and inter-tumor heterogeneity. **a** The UMAP of scRNA-seq data from a total of 12 ACPs. Cell clusters are denoted by color, and labeled with inferred cell types (top). The UMAP are also colored by the mutational status of the *CTNNB1* exon 3 in each cell (bottom left, gray dots for the cells with no reads covering the mutation loci, red dots for the cells with at least one read carrying the mutation bases [ALT], i.e., possibly mutated cells, and cyan dots for the cells with no alternative base found in the aligned reads [REF], i.e., likely the wildtype cells), and by the inferred cell cycling phase (bottom right). The circled clusters are the cycling cells (in G2M/S phases) with strong proliferating capability and they are found in the tumor cells, immune myeloid cells and T cells, and stromal cells. **b** Heatmap showing the expression of marker genes in 500 randomly sampled cells per cluster and a few representative gene labels are highlighted (see Suppl. Table 2 for the gene list). **c** Proportions of tumor cells, B cells and stromal cells among 12 ACPs, which reveals that there exist three 3 subgroups of ACPs. UMAP, Uniform Manifold Approximation and Projection; scRNA-seq, single-cell RNA sequencing; ACPs, adamantinomatous craniopharyngiomas; NK, natural killer; DCs, dendritic cells; cDC1, conventional type 1 DC; mDCs myeloid DCs; pDCs, plasmacytoid DCs

After rigorous quality control, the transcriptomic profiles of a total of 70,682 cells were obtained. We were able to identity 15 major cell types based on their expression of known marker genes (Fig. 1 and Suppl. Table 2). These cell types can be grouped into six main categories (1) tumor cells; (2) myeloid immune cells (including various dendritic cells [DCs], conventional type 1 DC [cDC1], myeloid DCs [mDCs] and plasmacytoid DCs [pDCs]); (3) lymphoid immune cells (T cells, NK cells, mast, plasma and B cells); (4) pituitary cells; (5) neural cells; and (6) endothelial cells and other stromal cells.

Whole-exome sequencing (WES) of the tumors and the matched blood samples was applied to determine the somatic variants in each tumor. Bulk RNA sequencing profiles were also available for 8 out of the 12 tumors. All the ACPs carried a driver mutation in *CTNNB1* exon 3, called from either WES or bulk RNA-seq data. The mutations in a majority of the tumors (7/12) occur at the late phosphorylation codon (4 at Ser33) or the flanking codons (2 at Asp32 and 1 at Gly34), 3 tumors at Thr41, and 2 tumors at Ser37. The variant allele fraction (VAF) ranges from 0 to 0.424, estimated from the WES reads, indicating a diverse range of tumor cell purity. These variants were used to annotate the cellular mutational status of *CTNNB1* exon 3 in the scRNA-seq data. Using inhouse code, each cell was annotated either as wildtype (REF, all the reads in the cell carrying the reference bases), mutated (ALT, at least one read carrying the alternative bases) or unknown (no reads covering the variant loci). With this information, two clusters of tumor cells were unequivocally identified. The cells expressing the marker genes of whorl-like cluster (WC) cells obtained from RNA-sequencing of laser-capture microdissection specimens, such as inhibitors of WNT/β-catenin signaling pathway, *DKK4* and *NOTUM*, forms an isolated cluster, showing distinct expression patterns from the other tumor cells.

As it has been observed in bulk RNA profiles and DNA methylome profiles, significant intertumoral heterogeneity was observed in terms of distribution of different cell populations. Four specimens (P455, P432, P452, P420) have a high proportion of tumor cells (> 8% in the corresponding specimens), while B cells are mainly found in P431, P419, and P457 tumors (> 14% in the corresponding specimens) in which the stromal cells are mostly missing (< 5% in the corresponding specimens; Fig. 1c).

The cell-cycle analysis results indicate that there are highly proliferative cycling cell subsets, in which cells are in S and G2/M phases only, in the tumor cell population, myeloid cell population, T cell population, and stromal cell population. The cycling tumor cells are likely the source of tumor proliferation while the cycling immune cells are reactive to killing the tumor cells.

### Palisading epithelial cells consist of a proliferating subset and whorl-like clusters are not tumor stem cells

**Fig. 2:**
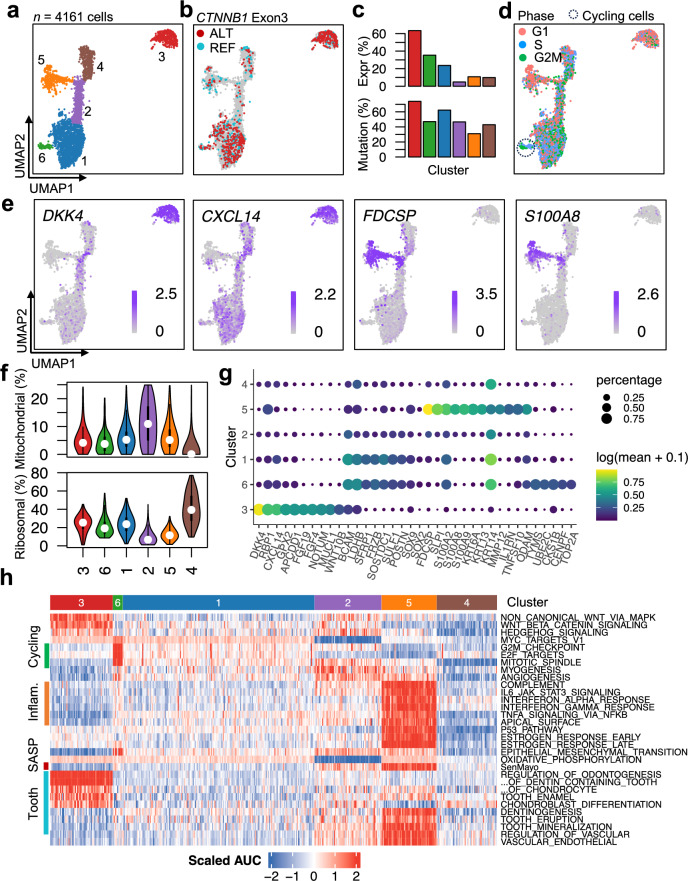
Subpopulations of the tumor cells. **a** The UMAP and re-clustering result of the tumor cells. Clusters are denoted by color. Cluster **3** is the whorl-like cluster tumor cells and cluster **1** is the PE cells. **b** The UMAP are colored by the mutational status of the *CTNNB1* exon 3 in each cell (the color and legend have the same meaning as in Fig. 1). **c** The proportion of cells with sequenced reads covering the *CTNNB1* exon 3 (top) and the proportion of mutated cells among the cells sequenced with reads covering the exon 3 (bottom). **d** The UMAP are colored by the inferred cycling phases. Cluster **6** is the cycling tumor cells (circled with the dotted line). (**e**) Dot plots showing the log-transformed expression of four representative marker genes. *DKK4* and *CXCL14* are mainly expressed in cluster **3**, and *FCSP* and *S100A8* are mainly expressed in cluster **5**. **f** Violin plots showing the distribution of the percentages of mitochondria reads (top) and ribosomal reads (bottom) in the six tumor clusters. **g** Bubble plots showing the log-transformed expression of representative marker genes for clusters **1**, **3**, **5** and **6**, where the size represents the percentage of cells expressing the genes and color hue represents the log-transformed mean expression levels. **f** Heatmap of the pathway enrichment values in each cell calculated with the scAUC algorithm for cancer hallmark pathways and tooth-related GO pathways

The cluster of the tumor cells other than the WC cells shows strong heterogeneity. In order to further characterize them, all the tumor cells were extracted and subject to re-clustering, which resulted in 6 subpopulations (Fig. 2 and Suppl. Table 3). The cluster **3** (T3) is the WC cells and the other tumor cells are divided into 5 clusters. The expression level of *CTNNB1* is the highest in T3 and decreases in the clusters **1** and **6** (T1 and T6) while the expression is very low in the other three clusters (T2, T4 and T5). In spite of the significant differences in the *CTNNB1* expression level among these subpopulations, the discrepancy is much smaller in the proportion of the mutated cells among the cells where *CTNNB1* exon 3 was detected, ranging from 31% in T5 to 74% in T3, which are not very far from 50%, the expected proportion of a heterozygous mutation, indicating that these cells are indeed tumor cells. T6 is the subpopulation of cycling cells and they express cell proliferation and division marker genes, e.g., *TYMS*, *UBE2C*, *CKS1B*, *CENPE*, *TOP2A* and *MKI67*. The cell-cycling hallmark pathways, such as ‘G2M_CHECKPOINT’, ‘E2F_TARGETS’ and ‘MITOTIC_SPINDLE’, are activated in this cluster. Other than these cell-cycle marker genes, the expression profile of T6 is similar to that of the T1 subpopulation. *SFRP1*, *FRZB* (also called as *SFRP3*)*,* and a few other genes that negatively regulate the WNT signaling are specifically expressed in T1 and T6.

**Fig. 3:**
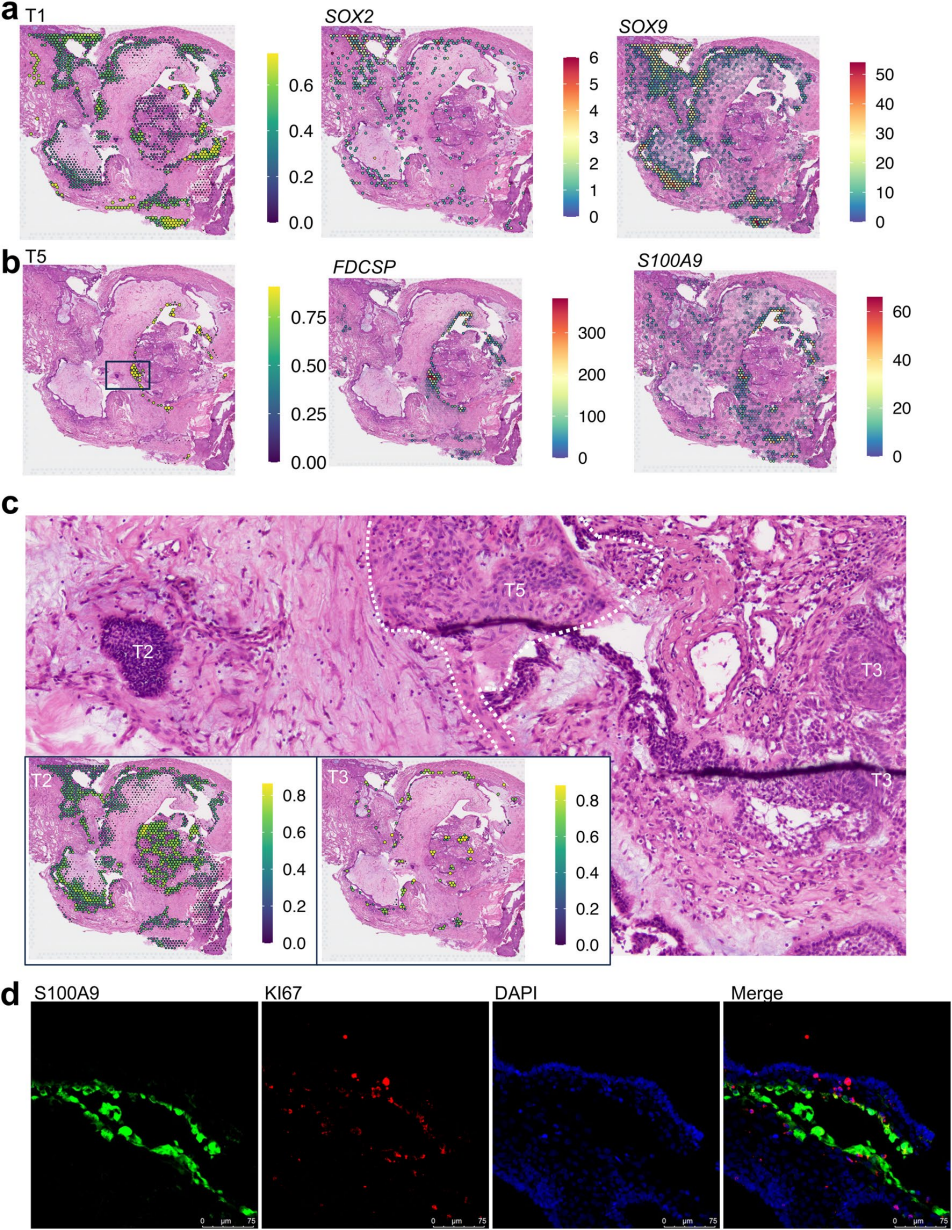
Spatial location of the subpopulations of the tumor cells. **a** Prediction scores for each spot in the Visium slide of P455 for cluster **1** of the tumor cells (T1) in the scRNA-seq data and the expression levels of *SOX2* and *SOX9* in each spot. **b** Prediction scores for cluster **5** of the tumor cells (T5) and the expression levels of *FDCSP* and *S100A9* in each spot. **c** The zoomed image of the square in the leftmost hematoxylin and eosin (H&E) staining image in (**b**) where representative tumor cells of T2, T3 and T5 are labeled. Prediction scores for T2 and T3 are shown on the bottom left. T3 are mapped to the whorl-like clusters and T2 to the densely packed tumor germinal centers. **d** Multiplex immunofluorescence imaging of S100A9, KI67, DAPI in an ACP specimen

To further identify these tumor cells, we applied the ‘anchor’-based integration workflow in the Seurat package to map the tumor subpopulations to spatial transcriptomic spots (Fig. 3). The results reveal that T1, the most abundant subpopulation, corresponds to the palisading epithelial (PE) cells and the adjacent squamous cells (those not orderly aligned as PE) as observed in the hematoxylin & eosin (H&E) staining images of the Visium slide from P455. The T3 subpopulation is mapped to the whorl-like cluster spots, confirming that T3 are indeed WC cells. The WNT/β-catenin and non-canonical WNT signaling pathways and hedgehog signaling pathway are activated in T3. Most of the cells in this subpopulation are in the G1 phase, without proliferating cells. Multiplex immunofluorescence staining also shows that Ki67-positive cells are distributed only in the PE cells, not found in the WC cells, indicating that proliferating tumor cells originate from the former not the latter. The genes *SOX2* and *SOX9*, which are commonly used to label stemness, are mainly expressed in the T1 subpopulation as well, with little expression in T3 (Suppl. Figure 1). Spatially, the two genes are also expressed in the PE spots only, not in the WC spots (Fig. 3). CD44 has been widely implicated as a cancer stem cell (CSC) marker, but it is widely expressed in the stromal and immune cells at a higher level than in the tumor cells. Although the expression level is higher in T3 than in T1, it is also expressed in T2 and T5 at a similar level (Suppl. Figure 1). This also indicates that in ACPs, the WC cells are unlikely the tumor stem cells.

### Senescent tumor cells are identified with specific expression of inflammatory factors and they have unique cell morphology

It has been hypothesized that the seeding cluster cells in ACPs drive the formation of tumors through a paracrine mechanism by secreting inflammatory and growth factors to form a senescence-associated secretory phenotype (SASP) [9, 29]. Although the T3 cells express inflammatory cytokines, *e.g.*, *CXCL14*, and fibroblast growth factors, such as *FGF4* and *FGF19*, they have the lowest SASP enrichment score calculated with the SenMayo gene set [31]. This indicates that the WC cells are not the senescent cells either. Instead, we identified a novel subset, the T5 subpopulation, in which the SenMayo gene set are enriched (Fig. 2h). Many inflammatory factors, *e.g.*, *FDCSP*, *S100A2*/*8*/*9* and *IL1RN* are expressed in this subset only (Fig. 2g). FDCSP is a secreted protein expressed in follicular dendritic cells which specifically binds to activated B cells [22]. S100A8 and A9 encode proteins to form calprotectin which is a calcium- and zinc-binding protein and plays a prominent role in the regulation of inflammatory processes and immune response, such as neutrophil chemotaxis and adhesion [36]. Inflammatory hallmark pathways are activated in this subset, including the ‘Complement’ system, the ‘IL6_JAK_STAT3’ signaling pathway, the ‘TNFα_via_NFκB’ signaling pathway, and the interferon α and γ response pathways, indicating that this subpopulation exhibits an inflammatory phenotype.

**Fig. 4:**
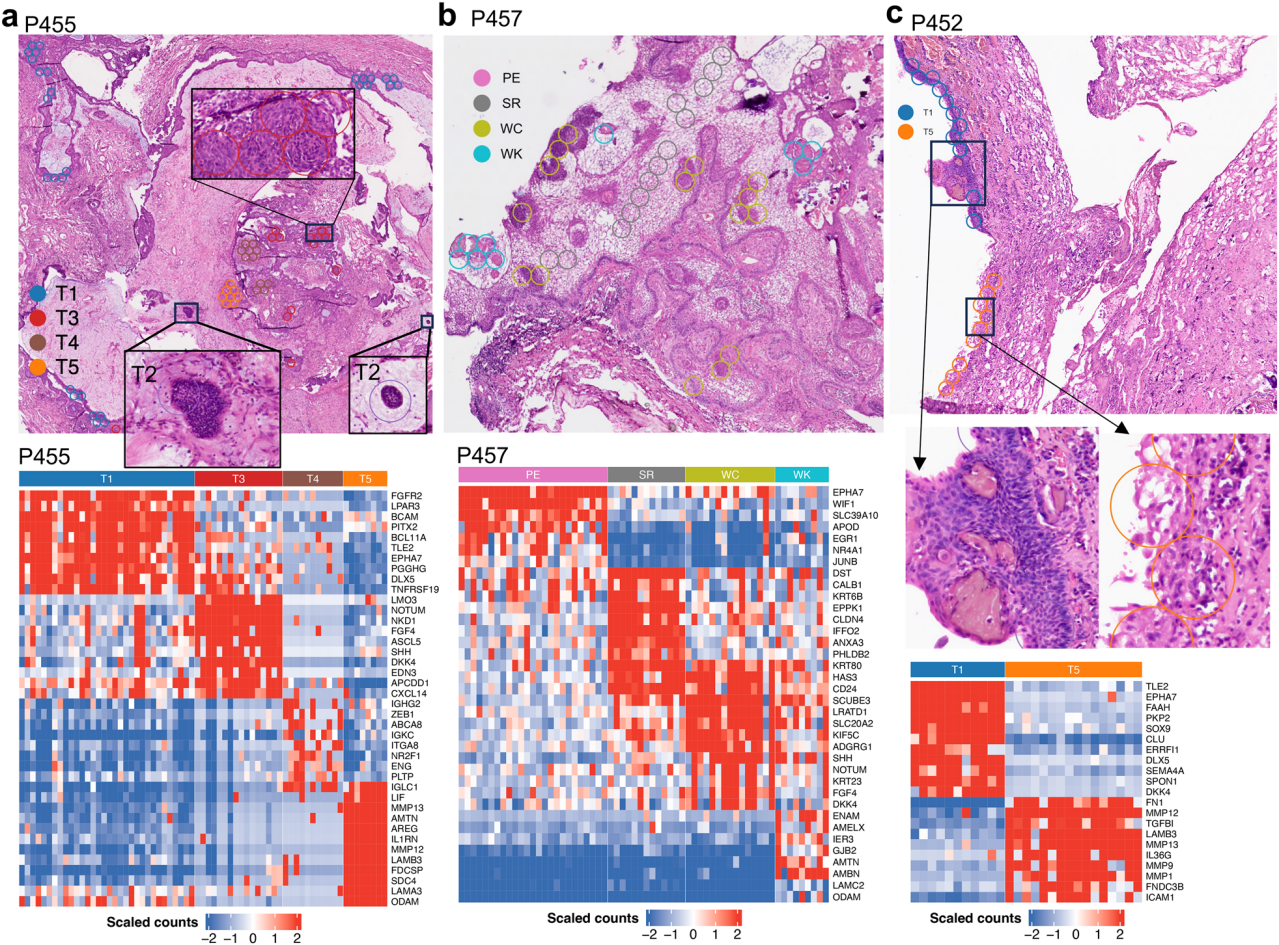
Molecular characteristics of manually selected spots in the Visium slides. **a** The selected spots which are annotated as T1, T3, T4 and T5 in the slide of P455 (top) and the scaled expression of the marker genes in each spot (bottom). Three zoomed images show the regions for one whorl-like cluster (T3) and two tumor germinal centers (T2). **b** The selected spots of four histological components, PE, SR, WC and WK, in the slide of P457 (top) and the scaled expression of the marker genes in each spot (bottom). **c** The selected spots which are annotated as T1 and T5 in the slide of P452 (top) and the scaled expression of the marker genes in each spot (bottom). Two zoomed images show the regions for one WC region surrounding WK along a thin layer of PE (left) and the SASP cells (T5). The center of each circle is located at the center of the corresponding spot and the diameter is 100 µm (inter-spot distance)

On the Visium spatial slide of P455, T5 cells were *not* mapped to the usual PE or WC spots, but instead to the loose squamous epithelial cell region (Fig. 4) where *FDCSP* and *S100A8*/9 are expressed. From the H&E staining image, it can be seen that T5 shows different morphological features from the usual PE or WC cells. Their nuclei are elongated to a narrow rectangle and they are not arranged in order. Immunofluorescence staining also found that S100A9 positive cells are at different regions from the PE cells (Fig. 3). In addition to the inflammatory pathways, estrogen signaling pathways are also activated. These results indicate that the cells in the T5 subpopulation are likely the SASP cells.

In the scRNA-seq data, T5 cells were found in all the tumors except in P433, and in 7 specimens there are more than 20 cells of T5 (Suppl. Table 3). As a comparison, only in P432 and P455 there are more than 10 cells of T3. On the spatial slides of the other two samples, the unique regions of T5 cells were also found (Suppl. Figures 2 and 3). These results suggest that, like PE cells, the senescent cells of T5 are ubiquitously present in ACPs.

### Single-cell and spatial sequencing also identifies tumor germinal cells

T2 is the second most abundant tumor cells. The scRNA-seq profiles of T2 and T4 subpopulations are similar as that of T1, and most of them were mapped to the adjacent region to T1 on the spatial slides. The proportion of mitochondrial genes in T2 is higher (median is 10%) than those in the other subpopulations, while the proportion of ribosomal genes in T4 is higher (median is 40%) than those in the other tumor cells (Fig. 2f). Therefore, like PE, they are the epithelial cells at different states.

In the Visium slide of P455, there are a significant number of tumor spots identified as T2 (Fig. 3). In particular, there are a few isolated spots with unique morphology in the stromal region with no other adjacent PE or squamous cells (Fig. 4). These cells are tightly packed together in a much higher density (approximately 1 cell per µm^2^) than other tumor cells. The size of each cell is small while the nucleus represents the majority of a cell, indicating that these burgeoning cells are the tumor germinal centers. Furthermore, the packing density is looser in the middle than that in the borderline suggesting that these germinal centers are expanding. Although the number of reads sequenced in these T2 spots are much smaller than those in T1 spots, the top-expressed genes in these T2 spots are similar with those in T1 spots.

**Fig. 5:**
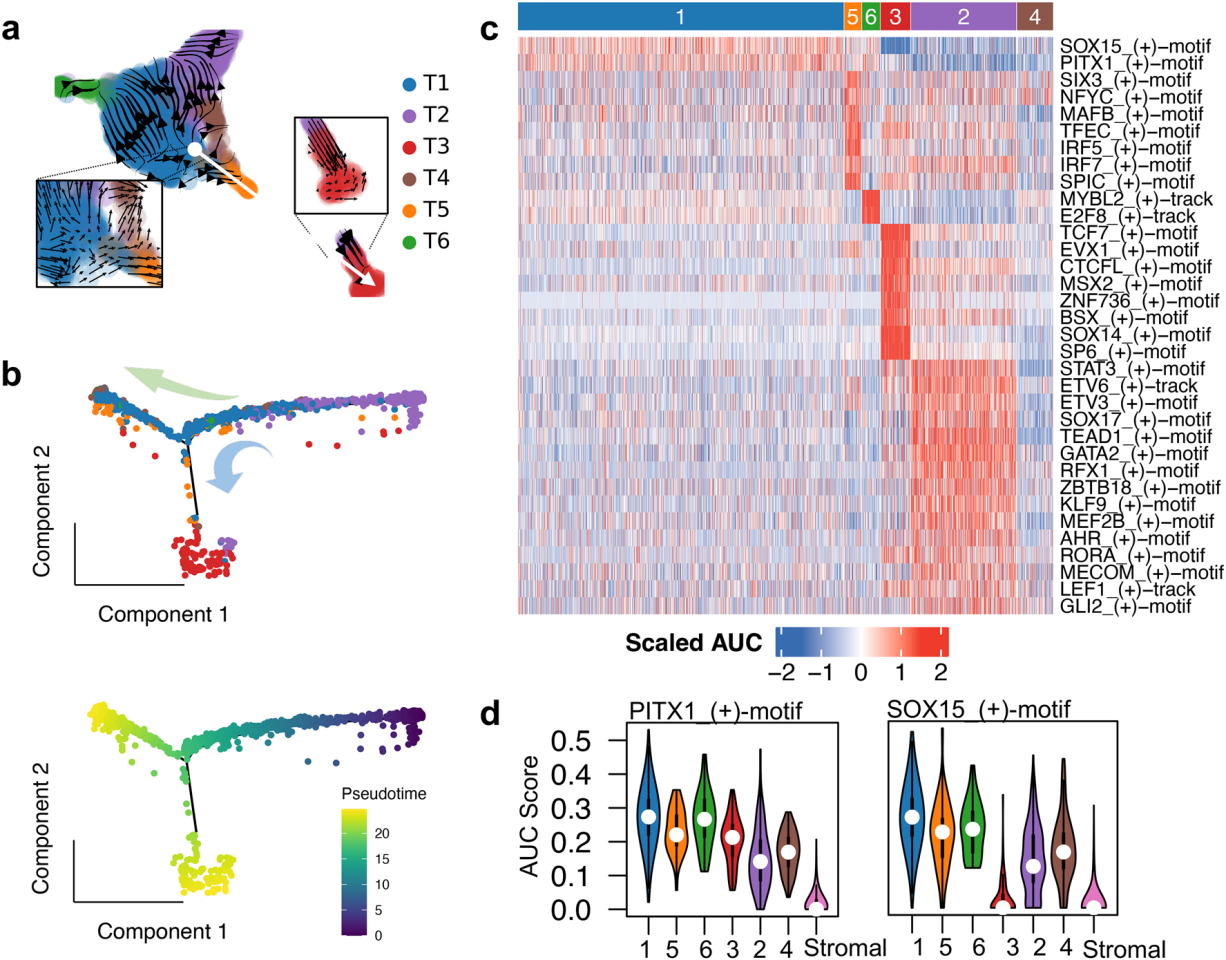
Developmental trajectories and transcriptional factor analysis results of tumor cells. **a** The embedded velocity streamlines in the RNA velocity analysis of the tumor cells in P455, showing major directions of cell progression in the transcriptional space. The zoomed regions show the source (left) and the drain (right) in the velocity field. The white arrow shows the trajectory from T1 to T3. **b** The trajectory of the tumor cells in P455 based on the pseudo-time analysis in Monocle2. The cells are colored by the cell cluster (top, the same color scheme as in **a**) and the pseudo-time (bottom). The arrows indicate the two trajectory branches. **c** The heatmap of the activity (scaled AUC scores) of the cluster specific regulons. **d** The violin plots of the AUC scores for two regulons, PITX1 and SOX15, in 5 tumor clusters. The plots for the stromal cells are also shown for comparison in which the two regulons are inactive

We applied the RNA velocity algorithm, scVelo, and the monocle pseudo-time analysis to infer the development trajectory of tumor cells in P455, the specimen in which there are abundant cells of all 6 subpopulations. Both algorithms revealed that there are two different development trajectories starting from the T2 cells (Fig. 5). Figure 5a shows the velocity streamlines, which describes the speed and the direction of the state transitions. One root state is found at the intersection between T1 and T2 clusters. Besides the major development trajectory in T1, there is another trajectory which ends in T3 (the white arrow). The monocle’s pseudo-time analysis also reveals a similar two-branch development trajectory. The trajectory starts from T2 and develops into T1 initially, and then branches into two fates, in which T3 is one terminal state, while the other terminal state consists mainly T1 and T5 cells. Similar results were also obtained in P432 (Suppl. Figure 4a). This result is consistent with that the T2 cells are the germinal tumor cells.

During the development process, the expression level of *CTNNB1* increases from the root to both the terminal states. Along the two branches of the trajectory, different sets of inhibitory genes of the WNT/β-catenin signaling are upregulated, *DKK4* and *NOTUM* in the branch to WC, and *SFPR1* and *FRZB* in the other branch (Suppl. Figure 4 and Suppl. Table 4). Different cytokines, e.g., *CXCL14* in one branch and *S100A10* in the other, are also associated with the development trajectories.

### Regulons of inflammation factors are activated in the senescent tumor cells

The SCENIC algorithm was used to determine the activity of regulons in the scRNA-seq data. The cluster-specific regulons are shown in Fig. 5c. Several inflammation-associated regulons are activated in T5, including interferon regulatory factors 5 and 7 (IRF5 and IRF7), NFYC which regulates the expression of major histocompatibility complex (MHC), and MAFB which is a critical regulator of complement component C1q. In WC (T3), the regulon of TCF7 is activated, and this regulon also acts as feedback transcriptional repressor of CTNNB1 besides forming a transcription complex with β-catenin. In T2, the activated regulons include LEF1, SOX17 and others. LEF1 is a key nuclear mediator of Wnt/β-catenin signaling and SOX17 inhibits Wnt signaling. The PITX1 regulon is activated in all tumor cells except that its activity is stronger in T1 (Fig. 5d) than in other subpopulations. PITX1 is a transcriptional regulator involved in basal and hormone-regulated activity of prolactin. The SOX15 regulon is activated in all tumor subpopulations except T3. This result suggests that targeting the transcription factors specific to the senescent tumor cells has the potential to inhibit their growth.

### Odontogenesis associated genes are expressed in the senescent tumor cells

Although in the scRNA-seq data the gene ontology (GO) biological process (BP) pathways of the regulation of odontogenesis, the regulation of chondrocyte proliferation and chondroblast differentiation, are activated in T3, the pathways of tooth eruption, tooth mineralization, regulation of vascular endothelial growth factor production and vascular endothelial growth factor production are activated in T5, not in T3 (Fig. 2h). Among odontogenesis related genes, *AMBN* is specifically expressed in T3, other genes including *ENAM*, *AMELX* and *AMELY* are not expressed in all the tumor cells.

Representative spots of T1, T3, T4 and T5, annotated with the scRNA-seq data were manually picked from the Visium slides to identify marker genes. As a comparison, representative spots of PE, SR, WC and WK were manually selected from the slide of P457, which are rich in various histological components, to identify marker genes in each of them (Fig. 4). From the expression heatmaps of the marker genes in Fig. 4, we can see that T1 are indeed PE cells with specific expression of *EPHA7*, *TLE2*, *WIF2*, *PITX2*, *FGFR2*, *EGR1*, etc. The T3 spots overlap with the WC regions, with characteristic expression of *NOTUM*, *DKK4*, *SHH*, *FGF4*, *KRT23*, etc. Besides a number of cytokines, e.g., *FDCSP*, *IL1RN*, *LIF*, other highly expressed genes in the T5 spots include some epidermal growth factors, e.g., *AREG*, proteinase *MMP13*, odontogenesis genes, e.g., *ODAM* and *AMTN*, and laminin genes, e.g., *LAMA3*, *LAMB3*, *LAMC2*. The odontogenesis associated genes, e.g., *ENAM*, *AMELX*, *AMTN*, *AMBN*, and the laminin gene *LAMC2* are expressed in the WK spots not in WC spots. These results suggest that senescent cells might play a critical role in odontogenesis while the WC cells regulates the process.

### Infiltration of immune cells and clonal expansion of cytotoxic T cells in the tumor microenvironment

**Fig. 6:**
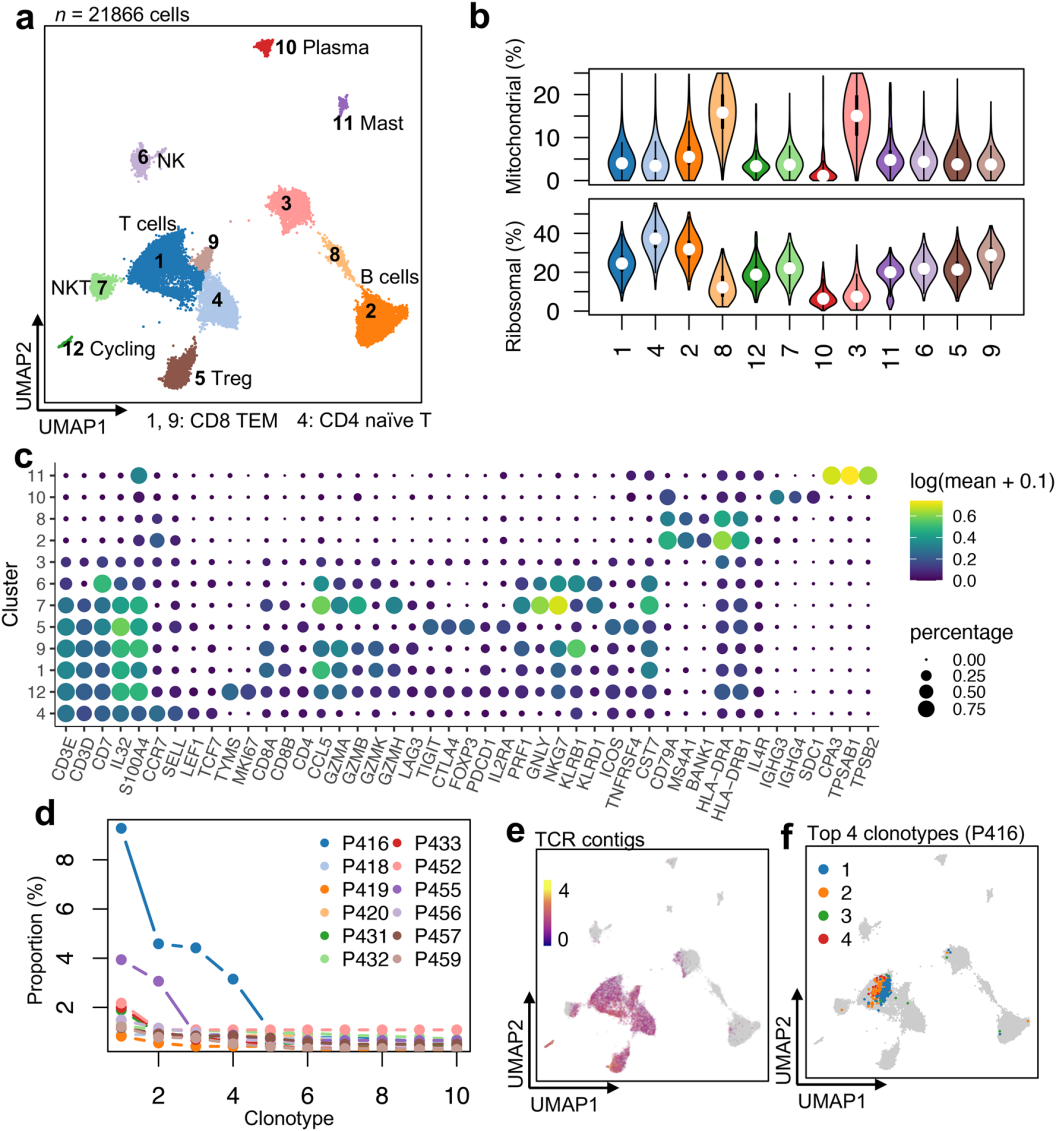
Subpopulations and clonal expansion of lymphocytes. **a** The UMAP of the re-clustering result of lymphoid cells, colored by the cell types. **b** Violin plots showing the distribution of the percentages of mitochondria reads (top) and ribosomal reads (bottom) in the clusters. **c** Bubble plots showing the log-transformed expression of representative marker genes, where the size represents the percentage of cells expressing the genes and color hue represents the log-transformed mean expression levels. **d** Clonal proportion of top 10 clonotypes of T cells of which the TCR was retrieved in each sample (see Suppl. Table 5). **e** The UMAP are colored by the number of TCR contigs in each cell. (**f**) The UMAP are colored by the top 4 clonotypes in P416 (clonotype 1, 168, TRA:CATGNSGNTPLVF, TRB:CASSLMGQAMGELFF; clonotype 2, 83, TRA:CAMRGIRSGGSNYKLTF, TRB:CSASPPGGVGANVLTF; clonotype 3, 80, TRB:CASSLMGQAMGELFF; clonotype 4, 57, TRB:CSASPPGGVGANVLTF). TCR: T cell receptor; CDR3, complementarity-determining region 3

In the scRNA-seq data, a total of 21,866 lymphoid immune cell expression profiles were obtained. Cluster analysis identified 12 subclusters, including T cells, natural killer T (NKT) cells, natural killer (NK) cells, B cells, mast and plasma cells (Fig. 6). A subcluster of cycling T cells was found, indicating the presence of rapidly proliferating T cells. Other T cell subpopulations include CD8 T effector memory cells (TEM), regulatory T cells (Treg) and CD4 naïve T cells. *GZMK* is highly expressed in CD8 TEM (clusters **1** and **9**) and the expression levels of *GZMB* and *GZMH* are higher in NKT (cluster **7**) than in CD8 TEM while *GZMA* and *CCL5* are expressed in both the CD8 TEM and NKT subpopulations. The results show that they are the cytotoxic effector cells. Tregs (cluster **5**) show high expression of *TIGIT*, *CTLA4*, *FOXP3*, *ICOS*, *IL2RA* and *TNFRSF4*.

We further integrated the scTCR-seq data with the scRNA-seq results. Although most of the clonotypes are singletons, *i.e.,* detected in only one cell, there are a few clonotypes in each specimen showing a high level of clonal expansion, particularly in P416 and P455 (Fig. 6d, and Suppl. Table 5). Most of the TCR contigs are found in the T cells, supporting the correct assignments of the T cells. We colored the cells carrying with the four largest TCR clonotypes of P416 in Fig. 6f. A majority of them are located in the CD8 TEM subpopulation, suggesting that these clonal-expanded cells are indeed cytotoxic T cells.

**Fig. 7:**
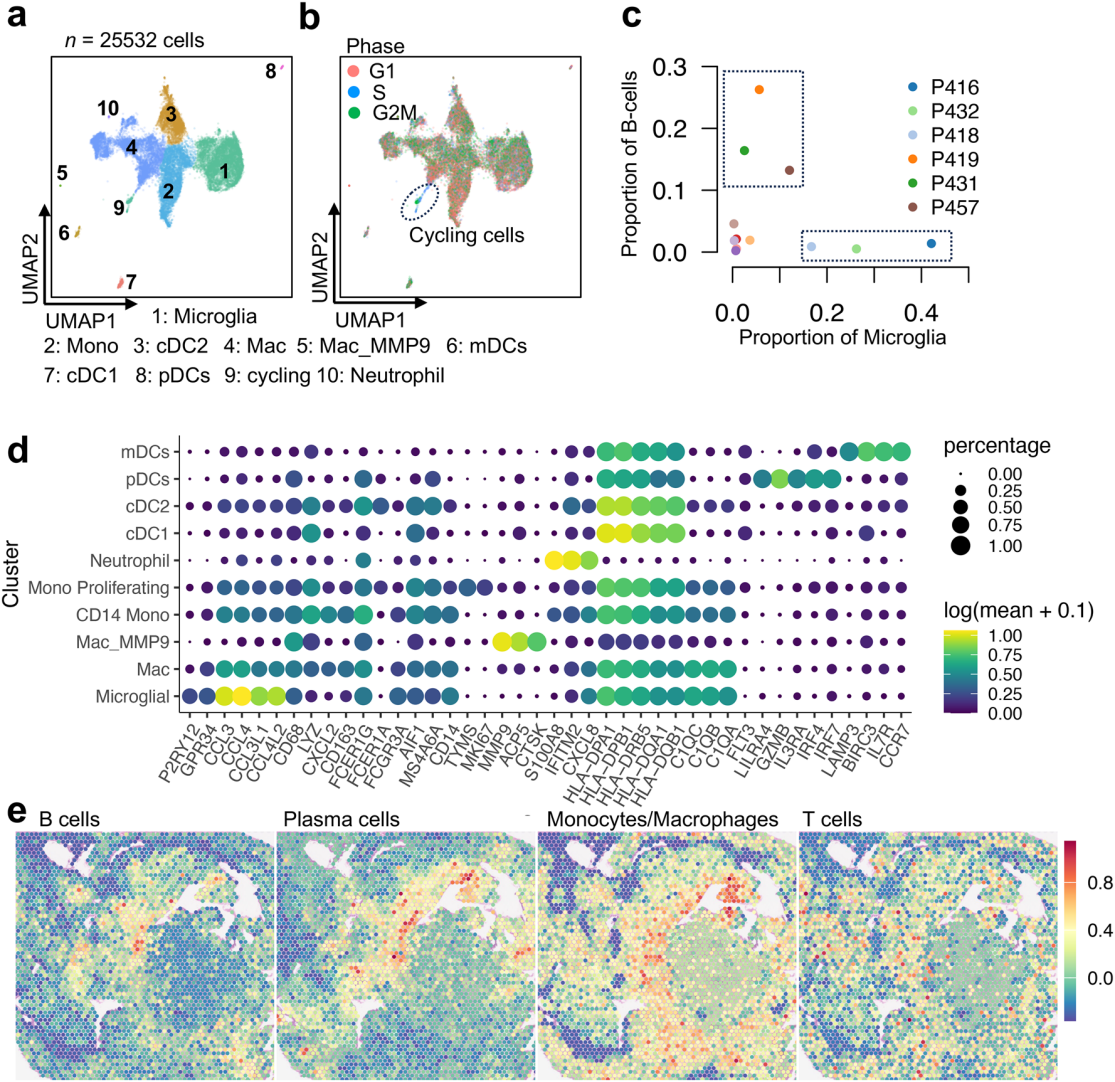
Subpopulations of myeloid cells. **a** The UMAP of the re-clustering result of myeloid cells, colored by the cell types. **b** The UMAP colored by the inferred cell cycle phases. The circled subpopulation is the cycling cells in the G2M/S phases. **c** Scatter plots of proportions of B cells versus those of the microglial cells in 12 ACPs. The 3 samples in the bottom right square are microglia dominant tumors and the 3 samples in the top left square are the B-cells dominant tumors while both are absent in the other specimens. **d** Bubble plots showing the log-transformed expression of representative marker genes, where the size represents the percentage of cells expressing the genes and color hue represents the log-transformed mean expression levels. **e** Score distribution of B cells, T cells, plasma cells and monocytes/macrophages on the spatial slide of P455 (see Fig. 4a)

The scRNA-seq profiles of a total of 25,532 myeloid immune cells were obtained, in which the microglial cells are the predominating subpopulation, with specific expression of *P2RY12* and a higher-level expression of *GPR34* and chemokines, e.g., *CCL3*, *CCL4*, *CCL3L1* and *CCL4L2*, than other cells (Fig. 7). The other myeloid subpopulations include monocytes, macrophages, DCs and a few neutrophil cells. In monocytes there are a small cluster of cycling cells. The major histocompatibility complex (MHC) class II molecules are expressed at a higher level in DCs than in microglial cells or monocyte/macrophages, but the CD1 family genes (*C1QA*, *C1QB* and *C1QC*) are mainly expressed in microglial cells, monocytes and macrophages, and the expression levels are higher in microglial cells and macrophages than in monocytes. The CD1 molecules are known presenting a broad range of lipid, glycolipid and lipopeptide antigens.

We notice that there is a mutual exclusion between B cells and microglial cells in ACPs (Fig. 7c). Most of the microglial cells come from P416, P432, and P418 tumors, in which the proportion of B cells is very low. In P419, P421, and P457, there is high abundance of B cells, but the proportion of microglial cells is very low. In the other six tumors, both cell types are relatively lacking.

The spatial distributions of several major types of immune cells in P455 are illustrated in Fig. 7e. Besides the accumulation of monocytes/macrophages in the inflammatory stromal regions, we observe that B, T and other immune cells cluster around the T5 cells. There is also clustering of these immune cells adjacent to the T1 region, whereas the scores for these immune cells are notably low in the spots adjacent to the T3 cells. A few spots with high T-cell scores are scattered among the T1 spots. These results suggest that the SASP cells possess the ability to recruit immune cells, whereas the WC cells lack such capability.

### Comparison between the primary and relapsed tumors

**Fig. 8:**
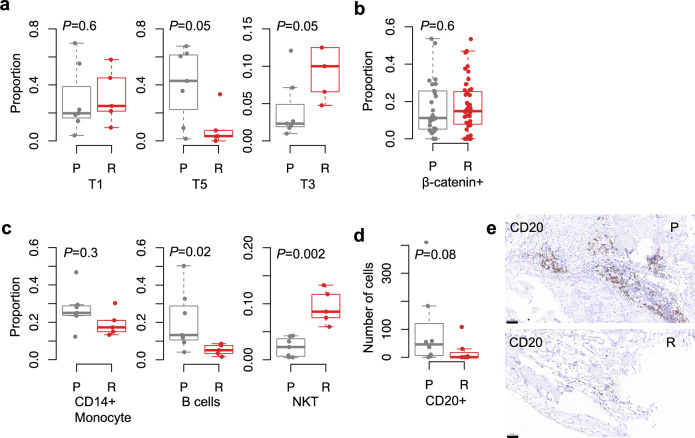
Comparison of tumor microenvironment between the primary (P) and relapsed (R) ACPs. **a** Box-whisker plots of the proportions of three tumor subpopulations in the scRNA-seq dataset grouped by the tumor status (P vs. R). **b** Box-whisker plot of the proportions of nuclear β-catenin positive tumor cells. **c** Box-whisker plots of the proportions of monocytes, B cells and NKT cells in the scRNA-seq dataset. **d** Box-whisker plot of the numbers of the CD20 + cells. **e** Typical IHC images of CD20 staining in primary (top) and relapsed (bottom) tumors. Scale bar, 50 μm. The *P* values were calculated with the Wilcoxon rank sum test

Among the 12 ACPs studied, 7 are the primary tumors and the remaining 5 are the relapsed tumors. Although an increasing trend is observed in the proportion of T1 tumor cells in the relapsed tumors, this difference is not significant (Wilcoxon’s *P* = 0.6, Fig. 8a). In contrast, the proportion of T3 cells is significantly higher in the relapsed tumors compared to the primary ones (Wilcoxon’s *P* = 0.05). The β-catenin staining results reveal that there is also an increasing proportion of nuclear positive cells, indicator of WC cells, in the relapsed tumors, but the difference is not significant (Wilcoxon’s *P* = 0.6, Fig. 8b). Additionally, we noted that the proportion of nuclear β-catenin positive tumor cells in the IHC results is significantly higher than that of T3 cells in the scRNA-seq dataset, reflecting the bias of single-cell sequencing technique which requires live tumor cells.

Contrary to T1 and T3, the proportion of T5 cells is significantly reduced in the relapsed tumors (Wilcoxon’s *P* = 0.05). This observation may stem from the fact that T5 cells are at a later developmental stage, rendering their proliferation rate less as rapid compared to T1 cells.

Among the immune cell populations, the proportion of B cells is significantly lower in the relapsed tumors compared to the primary tumors (Wilcoxon’s *P* = 0.02, Fig. 8c) while the proportion of NKT cells is significantly higher (Wilcoxon’s *P* = 0.002). IHC staining against CD20 supported the trend of the B cells (Wilcoxon’s *P* = 0.08, Fig. 8d, e). Additionally, the infiltration of T cells is also lower in the relapsed tumors although the difference is not significant (Suppl. Figure 5). These findings are in line with the observation of a lower proportion of T5 tumor cells in the relapsed tumors, suggesting potential alterations in the immune landscape during tumor relapse.

## Discussion

In this study, we delineated a full cellular landscape of different histological components in human ACPs at the single-cell level, pinpointed their positions with the spatially resolved transcriptomics sequencing and associated the molecular characteristics with the cellular morphology as observed in histological images. Although the total number of tumor cells is limited, we were still able to recapitulate the molecular features of these neoplastic cells at different developmental stages. The study identifies two novel subpopulations of the tumor cells, senescent and germinal cells, which display distinct molecular characteristics and cellular morphology. Our results exclude the WC cells being the stem cells or the SASP cells [9]. Indeed, the WNT/β-catenin signaling pathway and the sonic hedgehog (SHH) signaling pathway are activated in the whorl-like clusters, but only a limited number of cytokines and growth factors are highly expressed in these WC cells. The fibroblast growth factors that are specifically expressed in WC include *FGF19* and *FGF4* and the most prominent cytokine is *CXCL14*, which displays chemotactic activity for monocytes [15, 26]. The signature enrichment also shows that WC lacks the senescence-associated secretory phenotype. Stem cell markers *SOX9* and *SOX2* are not expressed in these cells either. Furthermore, RNA velocity analysis identified WC as a terminal state, not a root state. These results suggest that whorl-like clusters are not senescent stem cells in ACPs.

β-Catenin is a multitasking protein, both as the key nuclear effector of canonical WNT signaling in the nucleus and as an integral structural component of cadherin-based adherens junctions [7, 27, 35]. Its expression level is the highest in the WC subset and often shows nuclear accumulation. However, the transcription activation of the WNT signaling should be repressed due to the high expression of multiple inhibitors of the pathway, e.g., *NOTUM* and *DKK4*. Notum inhibits the WNT signal transduction by interacting with the ligands Wnts and DKK4 binds to LRP5/6 or further forms a complex with Krem-1/2 to induce endocytosis [35]. The structural function in adherens junction might be well preserved which acts through the armadillo domain. It is reasonably to hypothesize that the formation of the unique whorl-like morphology is due to excessive amount of β-catenin. The hedgehog pathway might also play a role in the relocalization of β-catenin from the cell membrane to the nucleus [18].

Instead of the whorl-like clusters, the SASP cells are those loose squamous epithelial cells without the ordered structures as in PE, which suggests that there lacks a strong inter-cell connection between these tumor cells. Many cytokines are expressed in these cells, including *FDCSP* and *S100A8*/*A9*. The former is often expressed in follicular dendritic cells and the protein is thought promoting cancer cell migration and invasion, and the latter induce neutrophil chemotaxis and adhesion [22, 36, 41]. The antagonist to interleukin 1 pathway *IL1RN* and tumor necrosis factor ligand *TNFSF10* are also specifically expressed in these cells. These cytokines are similar to those found in the cystic fluid in ACPs [3]. A number of keratin genes, *KRT6A*/*B*/*C*, *KRT7*, *KRT13* and *KRT17* are only expressed in the inflammatory-phenotype cells not in other tumor cells (Suppl. Figure 6). Proteinase *MMP12* and protease inhibitor *SLPI* are also only expressed in them. These results suggest that this subset are precursors to the formation of the cystic components in ACPs.

The second most abundant tumor cells following the PE subset are those we considered as tumor germinal centers. The reasoning is based on two results. One is that the pseudo-time analysis revealed that they are the root cells in P455. These cells have the highest expression level of mitochondrial genes and the lowest expression level of ribosomal genes among tumor cells. The other is that these cells are mapped to the densely-packed tumor cells. The size of these cells is small and the volume is dominated by the nucleus. Two isolated clusters are identified in the Visium slide of P455 and one is very small, approximately 50 µm in diameter, and in an elliptic shape. The other cluster is larger and the shape is no longer regular. The cell density of the outmost layer is higher than that in the center suggesting the clusters are expanding. But the cells in the outer layer are neither elongated nor well-aligned yet as PE. The other cells of this subset are those adjacent to the typical PE region. All these results indicate that this subset are the tumor germinal cells. Other than the two isolated clusters, there should exist smaller-size germinal centers, but which cannot be identified due to the limited resolution of the Visium spatial sequencing.

The most abundant tumor cells in our scRNA-seq data are the PE cells and adjacent squamous epithelial cells. Among them there is a subset of proliferating cells. Ki-67 positive cells are only observed in the PE region in our study and other studies. *SOX9* and *SOX2* are also specifically expressed in them. So are the Wnt antagonist, *SFRP1* and *FRZB*. Tumor suppressor gene *SULF1* is also specifically expressed which has been also found as a regulator of the WNT signaling [16]. The results are consistent with previous study and explain that PE is the most common histological feature in ACPs and they can be replenished with the proliferating cells. Based on the finding in this study, we proposed a hypothesis for the tumorigenesis and developmental trajectory. The seeding cells carrying the *CTNNB1* mutations gain the proliferation advantage due to the hyperactivation of the WNT/β-catenin signaling pathway and they become tumor germinal centers. As the WNT signaling is sustained for a prolonged time due that β-catenin cannot be degraded via the canonical pathway, two different negative feedback mechanisms are developed to repress the WNT signaling. One is via the upregulation of Notum and dickkopf family inhibitors and the other is via the upregulation of secreted frizzled related proteins. In the former scenario, the negative regulation is not so efficient in repressing the transcription of *CTNNB1* itself, excessive amount of β-catenin is still synthesized, and it leads to the formation of whorl-like clusters. While in the latter scenario, the transcription of *CTNNB1* is repressed and there is no much nuclear accumulation of β-catenin since it may be degraded via other noncanonical pathways independent of the phosphorylation of the degradation box. These cells develop into palisading epithelium, or squamous epithelium if β-catenin is inadequate to form tight junctions between cells. The whorl-like clusters eventually develop into wet-keratin nodules and squamous epithelial cells develop into stellate reticulum. Some of squamous cells develop into the SASP cells and they further develop into the cystic components. The hypothesis needs further experimental validation in the future.

Other than astrogliosis due to the finger-like infiltration of ACP tumors which has been described in the previous studies [3, 25], we identified cytotoxic T cells that target tumor cells via the integrated analysis of single-cell TCR sequencing with the scRNA-seq data. Among the lymphocytes in the tumor microenvironment, the cytotoxic T cells represent the most abundant subset. Strong TCR clonal expansion is found in at least 2 tumors. Therefore, the lymphocyte infiltration is not excluded due to the activation of the WNT/β-catenin signaling pathway as found in melanoma or other malignant tumors [19, 21, 33]. A subset of proliferating T cells is also identified indicating T cells can be replenished. Besides the cytotoxic T cells, regulatory T cells are found as well which may limit the anti-tumor immunity. Therefore, immune checkpoint blockade therapy may be applicable to treat some ACP patients.

One limitation of this study is that we obtained only more than 4,000 tumor cells although 12 ACPs were profiled. The reason is that the inter-tumor heterogeneity is large as revealed in previous studies. The choice of specimens was attempted to match their tumor status, age, subgroup and other factors with the general ACP population. Both the predominantly solid and predominantly cystic tumors were included. In some tumors there are abundant tumor cells such as in P455 while in others tumor cells comprise a small proportion of the cells in the microenvironment as in P452. For the spatial sequencing, we included 3 tumors which are representative for each subgroup in our previous study which shows that there exist three distinct molecular subgroups through analysis of bulk RNA sequencing and DNA methylation profiles [37]. Indeed, our study confirms that they are different from each other. The tumor of P452 grow on the pituitary stalk with a thin layer of PE and few SASP tumor cells. The tumor of P455 seems at the early stage of tumor trajectory with abundant germinal cells but few wet keratins or stellate reticulum. In contrast, the tumor of P457 is at the late developmental stage with a lot of wet keratins and stellate reticulum and the germinal cells are scarce. Another limitation lies in the potential introduction of biases during sample preparation and data acquisition for the current scRNA-seq approach, which may subsequently affect the results. Therefore, further experimental validation is necessary to verify the results obtained here.

In summary, we depict a comprehensive cellular map of diverse tumor cells in ACPs and reveal their developmental trajectories. Based on the findings in this study, we propose a mechanism for the formation of whorl clusters and palisading epithelium in ACPs which provide new ideas for therapy development.

### Supplementary Information

Additional file 1: **Table S1.** Basic information and *CTNNB1* mutation sites of 12 ACPs for single-cell RNA and TCR sequencing and 3 of them subject to spatial sequencing as well.

Additional file 2: **Table S2.** Marker genes for each cell type (Fig. 1b).

Additional file 3: **Table S3** **a** Marker genes for tumor clusters; **b** Numbers of cells each tumor cluster (T1–T6) in each specimen.

Additional file 4: **Table S4.** Gene significantly associated with the pseudo-time of the tumor developmental trajectory in P432 and P455.

Additional file 5: **Table S5** TCR clonotypes detected in each tumor specimen.

Additional file 6: **Figure S1.** Dot plots for the log-transformed expression levels of *CTNNB1*, *SOX2*, *SOX9* and *CD44* in the tumor cells.

Additional file 7: **Figure 2** Annotation of the tumor cells in the Visium slide of P457. **a** Prediction scores for cluster **1** of the tumor cells (T1). **b** Prediction scores for cluster **2** of the tumor cells (T2). **c** Prediction scores for cluster **3** of the tumor cells (T3). **d** The zoomed image of the squared region in (**a**). **e** Prediction scores for the inflammatory-phenotype tumor cells (T5). **f** The zoomed image of the squared region in (**e**). The cells circled with the dotted line are T5.

Additional file 8: **Figure 3** Annotation of the tumor cells in the Visium slide of P452. **a** Prediction scores for cluster **1** of the tumor cells (T1). **b** The zoomed image of the squared region in (**a**). Squamous epithelial cells surrounding PE are visible on the left and the pituitary stalk is on the right. **c** Prediction scores for T5, the inflammatory-phenotype tumor cells. **d** The zoomed image of the squared region in (**c**). Some of the T5 cells are circled out with the dotted line.

Additional file 9: **Figure S4** Developmental trajectory analysis results of tumor cells in P432 and the expression of the inhibitory genes of the WNT/β-catenin signaling pathway and two cytokines along the developmental trajectories. **a** The embedded velocity streamlines in the RNA velocity analysis, showing major directions of cell progression in the transcriptional space. The zoomed regions show the source (top right) and the drain (bottom left) in the velocity field. The white arrow shows the trajectory from T1 to T3. **b** The trajectory based on the pseudo-time analysis in Monocle2. The cells are colored by the cell cluster (left, the same color scheme as in **a**) and the pseudo-time (right). The arrows indicate the two trajectory branches. **c** The trajectory of the tumor cells in P455 inferred by the Monocle2 algorithm colored by the log-transformed expression levels of *FRZB*, *SFRP1*, *S100A10*, *DKK4*, *NOTUM*, and *CXCL14*, respectively. **d** The trajectory of the tumor cells in P432 colored by the log-transformed expression levels of the same gene set.

Additional file 10: **Figure S5** Comparison of tumor microenvironment between the primary (P) and relapsed (R) ACPs. **a** Box-whisker plot of the proportions of proliferating monocyte subpopulation in the scRNA-seq dataset grouped by the tumor status (P vs. R). **b** Box-whisker plots of the numbers of the IHC positive cells against CD3, CD38, MPO, CD68 and CD163. **c** and **d** Typical IHC images of CD30 staining in a primary (**C**) tumor and a relapsed (**D**) tumor. Four typical regions are shown for each slide. Scale bar, 50 μm. The *P* values were calculated with the Wilcoxon rank sum test.

Additional file 11: **Figure S6** Bubble plot of the keratin genes in the tumor cell clusters.

## Data Availability

Raw data reported in this paper has been deposited in the Genome Sequence Archive in National Genomics Data Center, China National Center for Bioinformation / Beijing Institute of Genomics, Chinese Academy of Sciences (GSA-Human: HRA006680) that are publicly accessible at https://ngdc.cncb.ac.cn/gsa-human.
