# Supplementary material for: Single-cell and spatial sequencing identifies senescent and germinal tumor cells in adamantinomatous craniopharyngiomas
Source: Cell Biosci. 2024 Sep 2;14:112. doi: 10.1186/s13578-024-01299-1 (PMC11370139; doi:10.1186/s13578-024-01299-1)
Supplement: Supplementary file 1 — Additional file 1: Table S1. Basic information and CTNNB1 mutation sites of 12 ACPs for single-cell RNA and TCR sequencing and 3 of them subject to spatial sequencing as well. [file 13578_2024_1299_MOESM1_ESM.docx]

**Suppl. Table 1** Basic information and *CTNNB1* mutation sites of 12 ACPs for single-cell RNA and TCR sequencing and 3 of them subject to spatial sequencing as well.

| PID | Sex | Age at diagnosis  (years) | Disease status | *CTNNB1* somatic mutation | | | | Spatial-seq |
| --- | --- | --- | --- | --- | --- | --- | --- | --- |
|  |  |  |  | Base | Codon | VAF in WES | VAF in RNA-seq |  |
| P416 | F | 14 | Primary | c.98C>G | p.Ser33Cys | 0.023 |  |  |
| P418 | M | 3 | Relapsed | c.97T>C | p.Ser33Pro | 0.223 | 0.293 |  |
| P419 | M | 17 | Primary | c.97T>C | p.Ser33Pro | 0.075 | 0.130 |  |
| P420 | M | 5 | Primary | c.122C>T | p.Thr41Ile | 0.136 | 0.105 |  |
| P431 | F | 50 | Primary | c.98C>G | p.Ser33Cys | 0.328 |  |  |
| P432 | M | 25 | Relapsed | c.122C>T | p.Thr41Ile | 0.424 |  |  |
| P433 | F | 13 | Relapsed | c.95A>G | p.Asp32Gly | 0.193 | 0.091 |  |
| P452 | M | 5 | Primary | c.101G>A | p.Gly34Glu | 0.104 | 0.518 | Yes |
| P455 | M | 3 | Relapsed | c.110C>G | p.Ser37Cys | 0.123 | 0.409 | Yes |
| P456 | F | 4 | Primary | c.110C>T | p.Ser37Phe | 0.239 | 0.423 |  |
| P457 | M | 42 | Primary | c.95A>G | p.Asp32Gly | 0 | 0.215 | Yes |
| P459 | M | 48 | Relapsed | c.122C>T | p.Thr41Ile | 0.117 |  |  |

ACP, adamantinomatous craniopharyngioma; VAF: Variant allele fraction; WES: whole-exome sequencing; PID: Patient ID; RNA-seq: bulk RNA-sequencing; Spatial-seq: spatial transcriptomics sequencing; Sex, F: Female, M: Male.
