# Supplementary figures and images for: Single-cell and spatial sequencing identifies senescent and germinal tumor cells in adamantinomatous craniopharyngiomas
Source: Cell Biosci. 2024 Sep 2;14:112. doi: 10.1186/s13578-024-01299-1 (PMC11370139; doi:10.1186/s13578-024-01299-1)

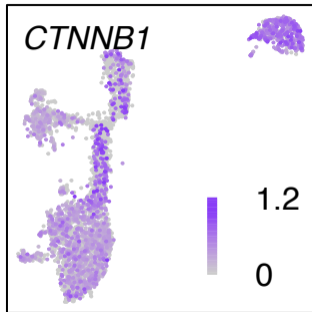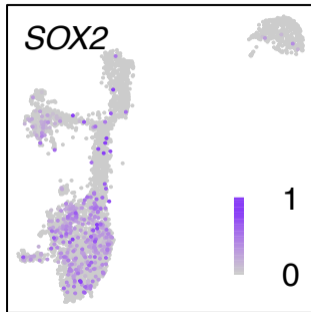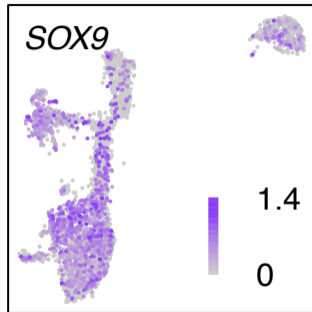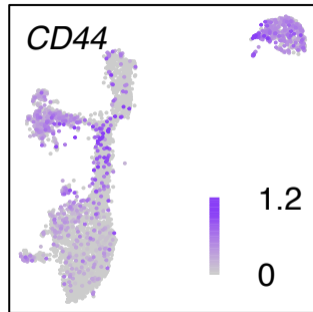

Supplement: Supplementary file 6 — Additional file 6: Figure S1. Dot plots for the log-transformed expression levels of CTNNB1, SOX2, SOX9 and CD44 in the tumor cells. [file 13578_2024_1299_MOESM6_ESM.pdf]

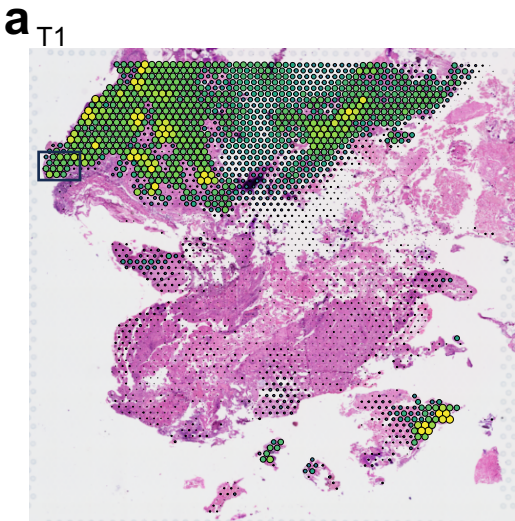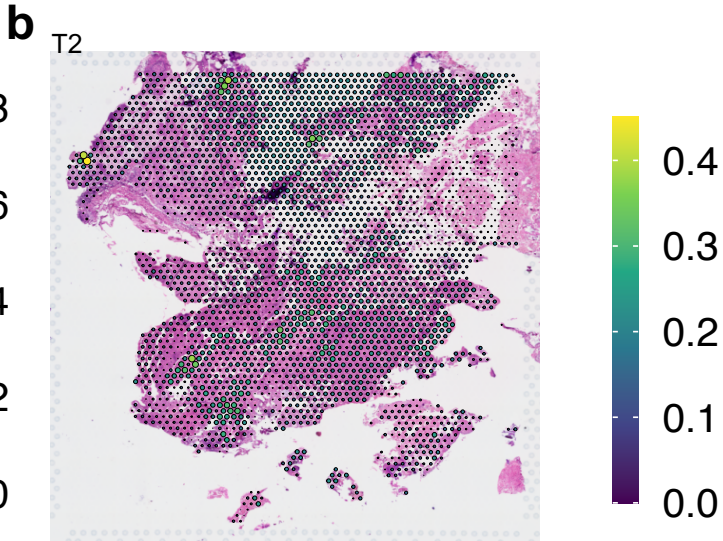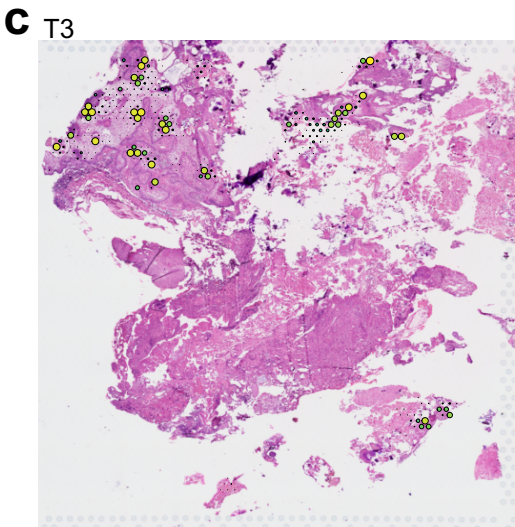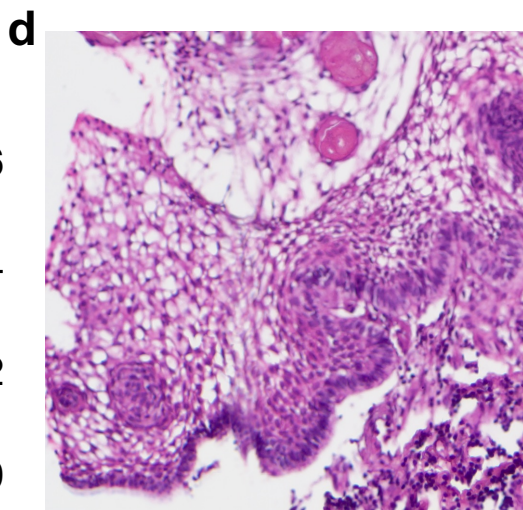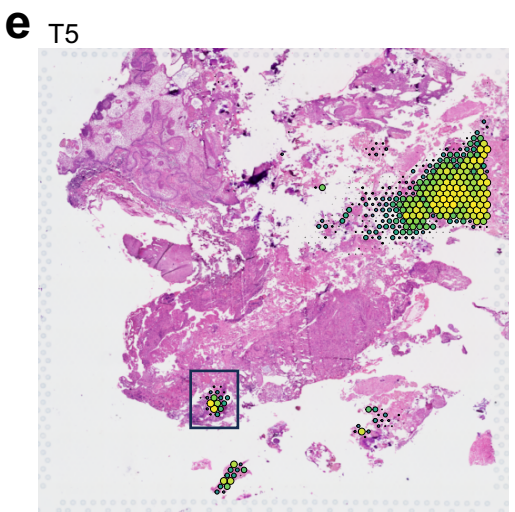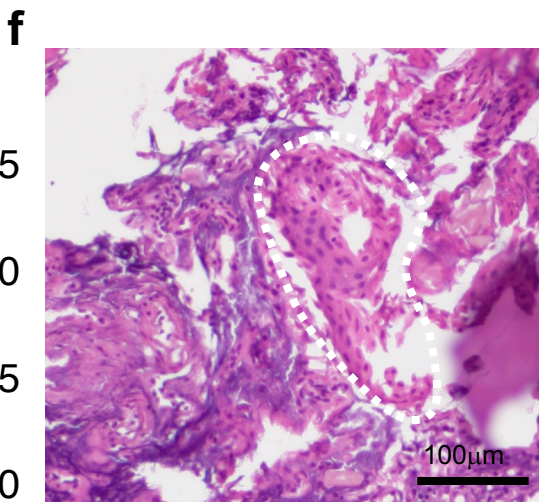

Supplement: Supplementary file 7 — Additional file 7: Figure 2 Annotation of the tumor cells in the Visium slide of P457. a Prediction scores for cluster 1 of the tumor cells (T1). b Prediction scores for cluster 2 of the tumor cells (T2). c Prediction scores for cluster 3 of the tumor cells (T3). d The zoomed image of the squared region in (a). e Prediction scores for the inflammatory-phenotype tumor cells (T5). f The zoomed image of the squared region in (e). The cells circled with the dotted line are T5. [file 13578_2024_1299_MOESM7_ESM.pdf]

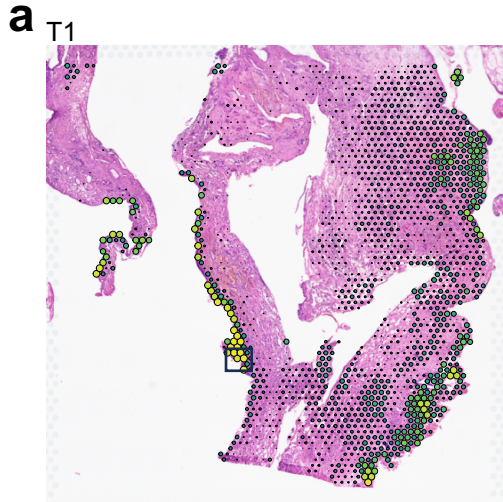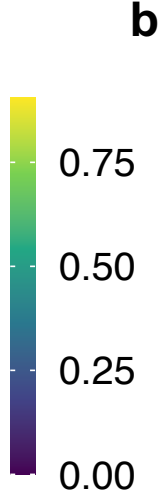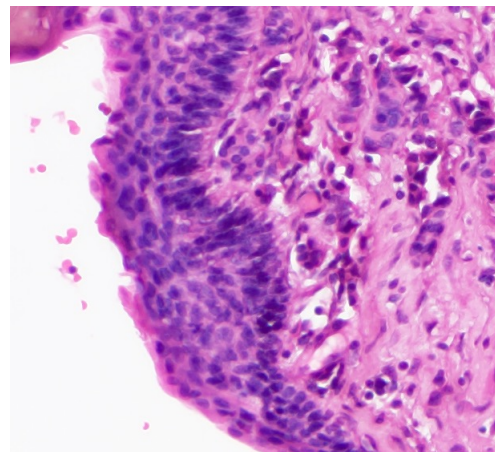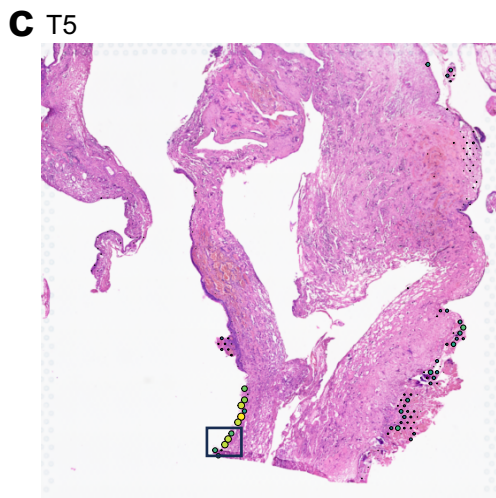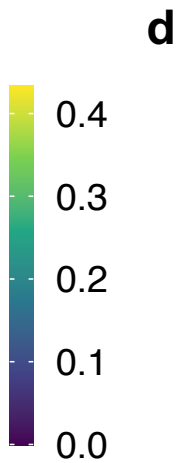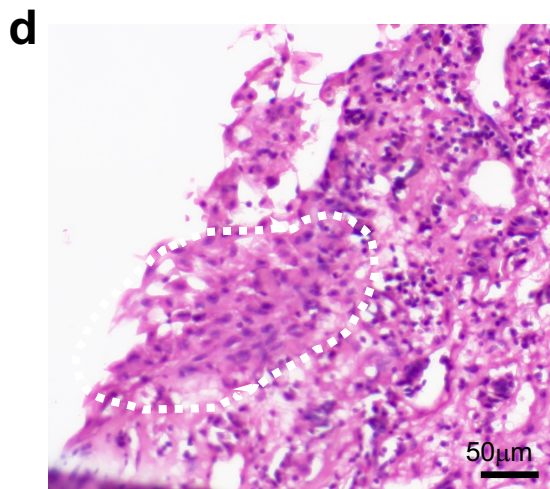

Supplement: Supplementary file 8 — Additional file 8: Figure 3 Annotation of the tumor cells in the Visium slide of P452. a Prediction scores for cluster 1 of the tumor cells (T1). b The zoomed image of the squared region in (a). Squamous epithelial cells surrounding PE are visible on the left and the pituitary stalk is on the right. c Prediction scores for T5, the inflammatory-phenotype tumor cells. d The zoomed image of the squared region in (c). Some of the T5 cells are circled out with the dotted line. [file 13578_2024_1299_MOESM8_ESM.pdf]

**a**

P432

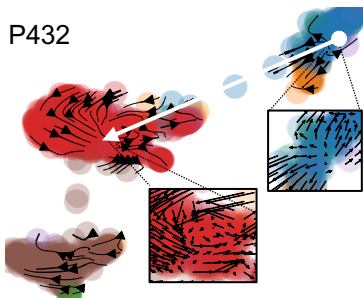

● T1  
● T2  
● T3  
● T4  
● T5  
● T6

**b**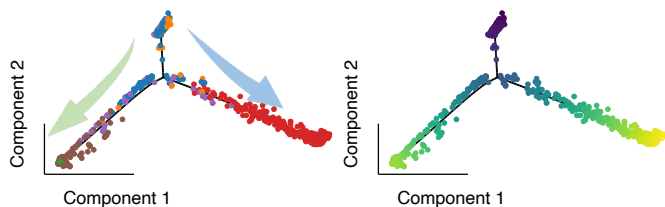**c**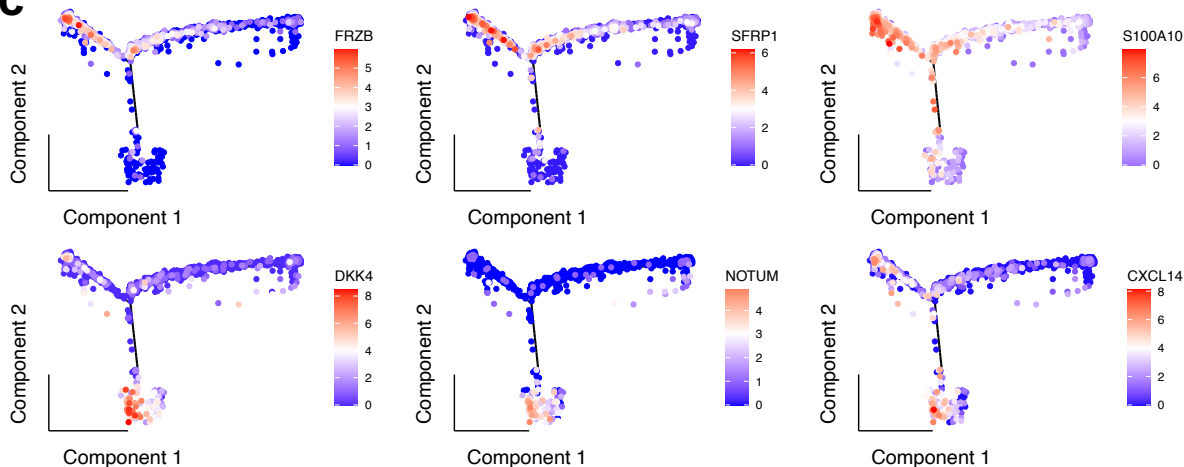**d**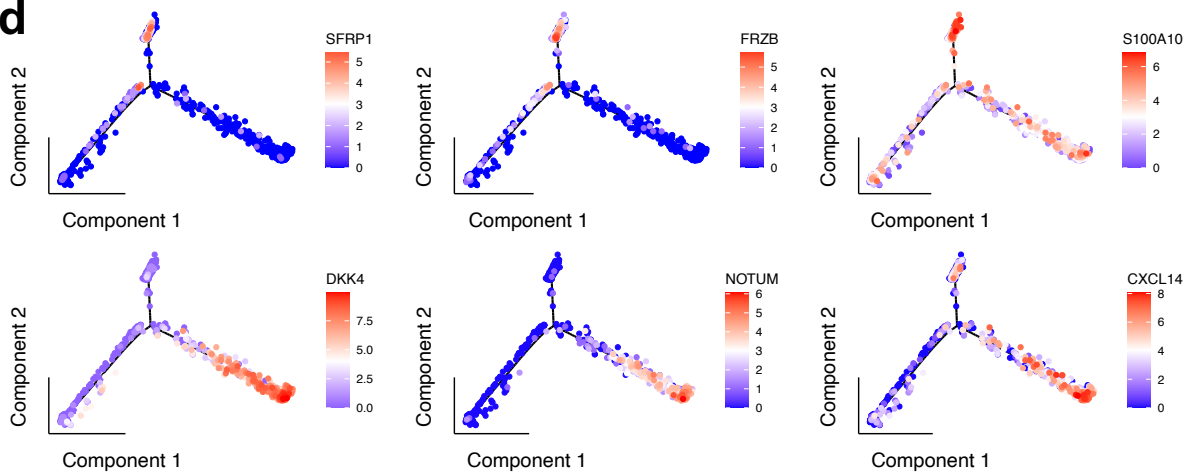

Supplement: Supplementary file 9 — Additional file 9: Figure S4 Developmental trajectory analysis results of tumor cells in P432 and the expression of the inhibitory genes of the WNT/β-catenin signaling pathway and two cytokines along the developmental trajectories. a The embedded velocity streamlines in the RNA velocity analysis, showing major directions of cell progression in the transcriptional space. The zoomed regions show the source (top right) and the drain (bottom left) in the velocity field. The white arrow shows the trajectory from T1 to T3. b The trajectory based on the pseudo-time analysis in Monocle2. The cells are colored by the cell cluster (left, the same color scheme as in a) and the pseudo-time (right). The arrows indicate the two trajectory branches. c The trajectory of the tumor cells in P455 inferred by the Monocle2 algorithm colored by the log-transformed expression levels of FRZB, SFRP1, S100A10, DKK4, NOTUM, and CXCL14, respectively. d The trajectory of the tumor cells in P432 colored by the log-transformed expression levels of the same gene set. [file 13578_2024_1299_MOESM9_ESM.pdf]

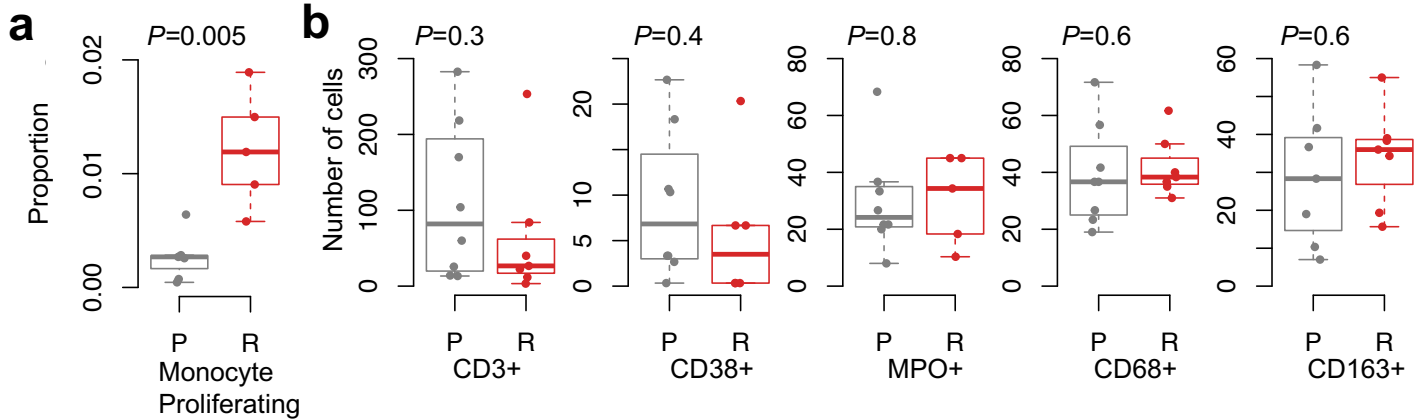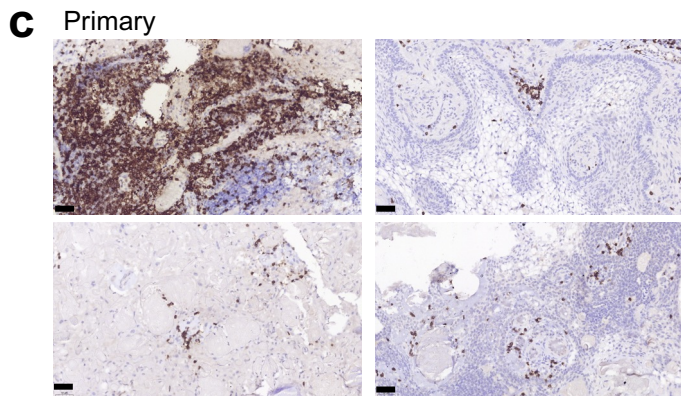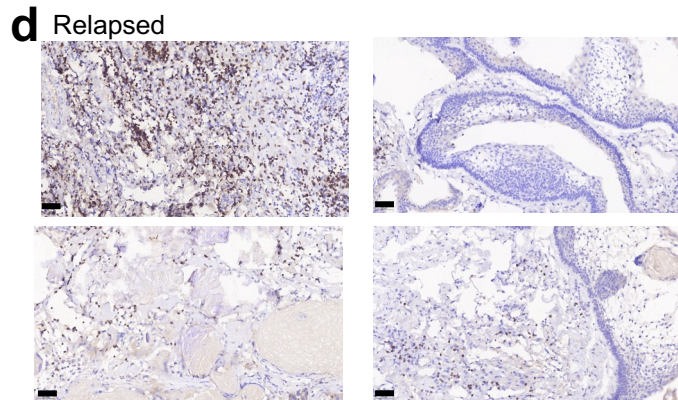

Supplement: Supplementary file 10 — Additional file 10: Figure S5 Comparison of tumor microenvironment between the primary (P) and relapsed (R) ACPs. a Box-whisker plot of the proportions of proliferating monocyte subpopulation in the scRNA-seq dataset grouped by the tumor status (P vs. R). b Box-whisker plots of the numbers of the IHC positive cells against CD3, CD38, MPO, CD68 and CD163. c and d Typical IHC images of CD30 staining in a primary (C) tumor and a relapsed (D) tumor. Four typical regions are shown for each slide. Scale bar, 50 μm. The P values were calculated with the Wilcoxon rank sum test. [file 13578_2024_1299_MOESM10_ESM.pdf]

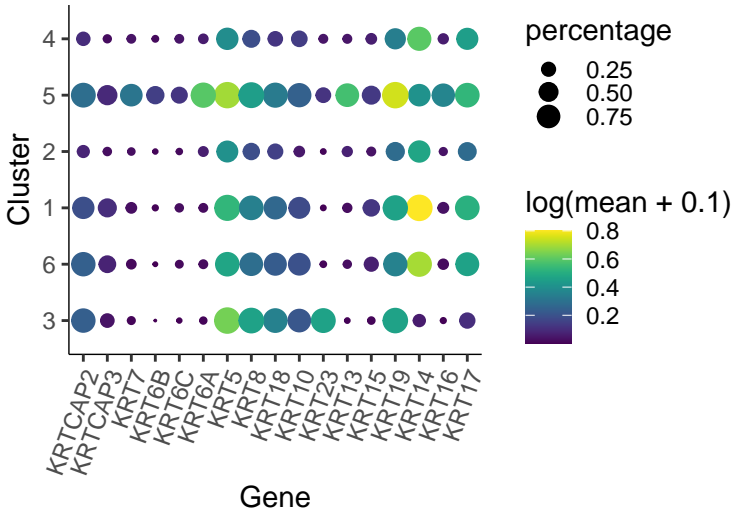

Supplement: Supplementary file 11 — Additional file 11: Figure S6 Bubble plot of the keratin genes in the tumor cell clusters. [file 13578_2024_1299_MOESM11_ESM.pdf]
